# Music@Home: A novel instrument to assess the home musical environment in the early years

**DOI:** 10.1371/journal.pone.0193819

**Published:** 2018-04-11

**Authors:** Nina Politimou, Lauren Stewart, Daniel Müllensiefen, Fabia Franco

**Affiliations:** 1 Department of Psychology, Middlesex University, London, United Kingdom; 2 Department of Psychology, Goldsmiths University of London, London, United Kingdom; Universitat Wien, AUSTRIA

## Abstract

The majority of children under the age of 5 appear to show spontaneous enjoyment of singing, being exposed to music and interacting with musical instruments, but whether variations in engaging in such activities in the home could contribute to developmental outcomes is still largely unknown. Critically, researchers lack a comprehensive instrument with good psychometric properties to assess the home musical environment from infancy to the preschool years. To address this gap, this paper presents two studies that describe the development and validation of the Music@Home questionnaire, which comprises two versions: Infant and Preschool. In Study 1, an initial pool of items was generated and administered to a wide audience of parents (*n* = 287 for the Infant, *n* = 347 for the Preschool version). Exploratory factor analysis was used to identify different dimensions comprising the home musical environment of both infants and pre-schoolers, and to reduce the initial pool of items to a smaller number of meaningful items. In Study 2, convergent and divergent validity and internal and test-retest reliability of the new instrument were established, using data from a different sample of participants (*n* = 213 for the Infant, *n* = 213 for the Preschool version). The second study also investigated associations between the Music@Home and musical characteristics of the parents, such as their musical education and personal engagement with music. Overall, the Music@Home constitutes a novel, valid and reliable instrument that allows for the systematic assessment of distinct aspects of the home musical environment in families with children under the age of 5. Furthermore, the Infant and Preschool versions of the Music@Home present differential associations with musical characteristics of the parents opening a new area of inquiry into how musical exposure and interaction in the home may vary across different developmental stages.

## Introduction

An increasing body of research has recently revealed beneficial effects of formal musical experience on early cognitive and linguistic development (e.g., [1–2]), with the majority of studies focusing on formal musical training given in classrooms (e.g., [3]). For most children under the age of 5 however, musical experience consists of everyday informal musical interaction in the form of singing songs, dancing, being exposed to recorded music and playing musical games in the home environment (for a review see [4]). This type of shared experience can potentially support learning, by providing a pleasant framework in which parents engage in activities with their children.

With respect to the effects that the home environment can have on children’s healthy development, ample and robust evidence has indicated that the amount and quality of language input in the early years is crucial for language acquisition [5–9]. A considerable body of literature has also demonstrated that the quality of the more general home environment, (conceptualized as everyday interactions and shared activities between children and their caregivers) can have beneficial effects on child development by providing more learning opportunities for children via increased frequency of activities, such as book reading and availability of learning materials. Positive developmental outcomes of enriched home learning environments include improved language and literacy skills [10], early numeracy skills [11–12], higher reading scores [13–14] and optimal social-emotional abilities [14–15]. Some measures of general shared activities have also included items related to music making (e.g. [15]) but very little research has focused on the musical environment at home per se. We suggest that the quantity and quality of informal musical activities may have a specific influence on the development of social, cognitive and linguistic abilities of the child, over and above influences emanating from a general enriched home environment.

One excellent example of informal musical experience introduced early in life is infant-directed (ID- henceforth) singing. ID-singing, similarly to ID-speech [16–17], can be distinguished from other types of singing mainly due to characteristics such as higher pitch, slower tempo ([18], mostly referring to lullabies), more expressive rendering of lyrics and higher emotional engagement [19–20]. Studies have shown that maternal singing can regulate the levels of arousal in 6-month-old infants [21] and infants show increased responsiveness (visual feedback) to videos of mothers singing versus speaking at 6 [22] as well as 11 months [23], presumably because mothers’ singing performances are rated as more emotional when compared to speech [22]. Studies have also indicated that singing facilitates speech segmentation in both infants [24–25] and adults [26] highlighting its potentially beneficial role in language development. Indeed, singing may support language learning through the presence of pitch variations that can aid in the discrimination of syllables [26] while also increasing interest and attention [25–26].

Parental singing may hold a central position in early musical interactions where infants’ activities are limited [23], [27–29], but the repertoire of musical activities becomes more varied as children grow older [30–33]. An elegant qualitative analysis of 18 young children’s recordings, parental diaries and interviews with caregivers, provided rich information about parent-child musical activities in the preschool years [34], which appear to include joint and supported singing (e.g., children’s songs, counting songs, and nursery rhymes), improvising songs to accompany everyday routines, dancing, playing musical (including toy) instruments, and listening to recorded music [34–35]. Critically, the majority of studies that have explored the home musical environment in the early years report that most parents of children under 6 years interact musically with their children in various ways [28–29], [31], [33]. Furthermore, children’s home musical activities appear to have increased in recent years, a tendency associated to the rapid advancements in digital technologies and to sociological factors such as children spending more time at home and less time playing outdoors [36].

Given the predominance and richness of musical activities within family environments and the spontaneous enthusiasm that the majority of young children show for musical play, it is surprising how little we know about the effects that this dimension of parent-child interactions can have on development. To our knowledge, two studies so far have directly addressed the effect of such informal musical experience (not limited to maternal singing), on early cognition and language: [37] and [38]. Putkinen and colleagues [37] asked parents to report how frequently their 2- and 3-year-old children engaged in activities such as singing and dancing as well as how often they interacted musically with their children. Higher scores in these reports were significantly related to more refined ERP responses associated with change detection in the duration and temporal structure of sounds. These results suggested that children whose environment was more musically enriched had developed more mature auditory processing at the neural level. Williams and colleagues [38] employed a longitudinal design to assess the effect of enriched musical activities in the home using a single-item measure in parent self-reports when their children were 2 and 3 years old, on cognitive, emotional and social markers of development 2 years later. Moderate associations were found between the frequency of shared musical activities at 2 and 3 years and the children’s vocabulary, arithmetic abilities, attentional and emotional regulation, and prosocial skills 2 years later. Critically, small effects (*β* < .08) of shared musical activities on all of the above outcomes were maintained even when the authors controlled for the effect of shared book reading, an activity found to be strongly associated with later academic achievements [10], [39].

Although these two studies were valuable in identifying associations between frequency of musical interactions and both auditory processing [37] and cognitive and socio-emotional aspects in early development [38], both used a small number of ad hoc frequency items (7 items in [37], 1 item in [38]) to describe musicality in families. However, the home musical environment can consist of a number of dimensions (e.g., parental singing, the child’s engagement with music) differentially relating to aspects of early development and a small number of items broadly evaluating musical experience in the home might fail to capture these associations. Perhaps for this reason some studies have produced inconsistent results. For instance, Hartas [15] conducted a longitudinal study to investigate the effects of the home learning environment on developmental outcomes in a sample of 15,600 3- to 5-year-old children. Two items embedded in parental interviews that assessed the frequency of singing songs/rhymes and playing music at home did not exhibit significant associations with later linguistic or social-emotional enhancements. It is worth noting however, that in this study there was no direct measurement of children’s socio-emotional and language and literacy development but rather results were based on teachers’ ratings.

A number of parent-report tools have been used in the past to assess parent-child musical engagement at home for different age groups. To our knowledge, the first parent self-report instrument designed to explore the musical environments of school-aged children, the Home Musical Environment Scale (HOMES), was published by Brand in 1985 [40]. The HOMES comprised 4 factors relating to different aspects of parent and child involvement with music (i.e., i. parents’ attitude toward music and musical involvement with child, ii. parental concert attendance, iii. parent-child ownership and use of records/tapes and iv. parent plays a musical instrument) and displayed good reliability and concurrent validity, as tested with a sample of 7-year-old children’s families. The main motivation for its development was for music educational purposes such as nourishing the musical potential of primary school students or exploring associations between the HOMES and children’s musical attainment [40–41]. The HOMES was not intended for the use with infants or preschoolers. Another validated instrument recently created to assess the musical behaviour of children under the age of 5, is the Children’s Musical Behavior Inventory or CMBI [42]. The CMBI is an 8-factor, 97-item parental report designed to assess the frequency by which children engage in a number of musical behaviours such as music-listening, singing and dancing, with the aim of identifying and meeting their musical needs in preschool education settings. While seven of the eight factors in the CMBI are associated with child-initiated musical behaviours one factor also assessed the frequency of parent-initiated musical activities. The CMBI dimensions showed good reliability (Cronbach’s α ranged from .77 to .97) and adequate construct validity as tested with confirmatory factor analysis [42].

Other studies have used ad-hoc questionnaires and/or parental interviews to explore how parents use music at home with children younger than 6 years, either for descriptive purposes [28], [30], [33], [35] or to examine how the parents’ previous experience with music can affect current musical engagement with their children [29], [31], [43]. All of the instruments used in the above studies explored musical experience in specific age groups focusing on either the infant or the preschool range.

Indeed, most of the home musical environment measurements used so far have addressed specific aspects of this experience such as frequency of musical interactions while neglecting others, such as breadth of musical exposure or parental beliefs regarding music and development. In addition, apart from the CMBI [42] there is no available validated measurement instrument for the younger age group. Therefore, we deemed it necessary to create a systematic, easily administered and brief research tool that would encompass a range of parent and child musical behaviours for children under the age of 5.

The aim of the present study was to develop a valid and reliable parent-report instrument that can be used for the assessment of the home musical environment from infancy to the preschool years and in research examining potential effects of this type of environmental experience on a range of developmental outcomes. Within the development of this instrument, another aim was to elucidate potentially different dimensions comprising informal musical experience in the home for different developmental levels, i.e., infancy and preschool years. To the above ends, Study 1 included the generation of a pool of items that were administered to a large sample of participants. Exploratory factor analysis was used to identify underlying dimensions within the items and reduce the initial pool of items to a smaller number. In Study 2, data from a different sample of participants was used to establish convergent and divergent validity and reliability of the new instrument. Furthermore, in Study 2 we assessed whether the Music@Home would be associated with musical background and behaviours of the parents, such as musical education and personal engagement with music. The research undertaken and presented in this paper received ethical approval from the Ethics Committee of Middlesex University as conforming to the ethical principles of the British Psychological Association and the WMA Declaration of Helsinki.

## Study 1: Development of Music@Home questionnaires

In the first study, two questionnaires were created: the Music@Home-Infant (3 months to 1 year and 11 months) and the Music@Home-Preschool (2 years to 5 years and 6 months) respectively, aiming to capture and quantitatively assess the range of musical behaviours occurring in the home environment of families with young children. Two years of age was taken as a cut-off between infancy and the preschool years for practical reasons: this instrument is envisaged to be used in research looking at the effects of the home musical environment on developmental outcomes, such as formal language and cognitive assessments. Beyond two years most children will have achieved major developmental milestones such as walking and finer motor control, symbolic thinking and talking. Although many children begin to produce multiword sentences before the age of 2, only in the third year are most children verbally fluent. Thus, before and after 2 years are stages that are likely to offer different opportunities for parents’ musical interactions with their children.

## Materials and methods

A total of 67 items were compiled covering a broad definition of musical experience at home, which was conceived as: “informal musical interactions and/or musical activities directed at or involving children”. Items were selected based on a review of relevant pre-existing items (Musical Experience in the Family Questionnaire; provided by co-author of this manuscript Fabia Franco, [31], [37], [38]), and on preparatory work conducted in the two years leading up to the start of the project. This involved informal conversations with parents of infants and preschoolers spanning personal circles and nurseries in four countries (UK, France, Italy, Australia and Greece). In addition, two families recruited through personal connections (a family of an infant and a family of a preschool child) agreed to write down short descriptions of any music-related activity that they may engage in with their children at home. Notes from informal conversations and written descriptions were subsequently used to compile a list of activities that formed the basis for item generation.

The initial 67-item list covered 12 different aspects of musical experience at home, which were considered by the team as relevant to the working definition. These dimensions were: Structured musical activities, Parental attitudes towards music, Music use for child’s emotional/mood regulation, Child listening to music, Child moving to music, Child music making, Infant and child directed singing, Child singing, Breadth of musical exposure, Daily routine and home activities, Child music preferences, Beliefs about music and development. Within each of the above dimensions we ensured as much as possible that negatively and positively worded items were balanced. The majority of items were applicable to both infants and preschoolers. However, 7 items relevant to preschoolers and deemed necessary in the description of informal musical experience in this age group, could not apply to infants (e.g., My child sings along to music on the television). Therefore, two versions of the questionnaire were created: an infant version containing 60 items, and a preschool version containing an additional 7 items. A 7-point agreement-disagreement scale was used for all items, ranging from Completely Disagree (1) to Completely Agree (7). Item scores for negatively worded items were inversely coded such that the score for Completely Disagree (1) corresponded to the score for Completely Agree (7).

### Participants

English-speaking participants (including advanced non-native, fluent and native speakers) from English-speaking and other countries were recruited via social media, parent networks, participant databases and public mailing lists. To be eligible for the study, participants had to be parents of children between the ages of 3 months and 5 years and 6 months. A total of 724 individuals (n = 395 for the Preschool and n = 329 for the Infant version) took part initially. However, 90 participants (*n* = 48 for the Preschool and *n* = 42 for the Infant version) were excluded for [i] having children outside the age range reported above (they were either under 3 months or over 5 years and 6 months), [ii] completing fewer than 80% of the questionnaire items.

A total of 347 participants were therefore included in the Music@Home-Preschool analysis. The primary caregiver completing the survey was predominantly the mother (n = 312), while in a few cases it was the father (n = 34) or another relative (n = 1). The mean age of participants was 36.56 years (*SD =* 4.78) and the mean age of the children for who the survey was completed was 3.58 years (*SD* = .98). Detailed demographic information for participating parents and children is reported in S1 and S2 Tables respectively.

A total of 287 participants were included in the Music@Home-Infant analysis. The primary caregiver completing the survey was predominantly the mother (n = 265) while in 22 instances it was the father who completed the survey. The mean age of participants was 35.13 years (*SD =* 5.29) and the mean age of the children for whom the survey was completed was 14.52 months (*SD* = 6.54 months). Demographic details for participating parents and infants are summarized in S3 and S4 Tables respectively.

### Procedure

The infants and preschool surveys were constructed using the *Qualtrics* online survey tool [44] and participants were directed to the appropriate version according to the age group of the child for whom they completed the survey (i.e., Infant or Preschool). The Music@Home questionnaire was intended for the primary caregiver (i.e., the caregiver that spends more time with the child); in case of equal parenting, either caregiver could complete the questionnaire. Participants were asked to take into account all musical interactions in the family that may involve the child, including activities that are carried out by other members of the family (e.g., carers or siblings).

Parents were invited to complete the survey by clicking on an online link, which was posted on social media and parent networks or emailed to participant databases and public mailing lists. Participants were first required to give their informed consent and then completed the Music@Home items along with a short demographic questionnaire. If taken without pauses, completion of the survey took approximately 20–30 minutes. Participants received no compensation for completing the survey.

### Statistical analyses

All data were analyzed using the R software environment [45]. Exploratory factor analysis (EFA) was employed in both infant and preschool versions of the Music@Home using the psych package [46] to identify different dimensions within the set of items that would correspond to subscales of the questionnaires. Two factor extraction methods typically used in exploratory factor analysis were implemented i.e., maximum likelihood estimation and minimum residual factor analysis. The criteria employed for factor extraction included: parallel analysis [47–48], Kaiser’s criterion (only factors with eigenvalues >1 are retained; [48], visual inspection of the screeplot [49], Velicer’s Minimum Average Partial (MAP) criterion [50], and Revelle and Rocklin’s Very Simple Structure (VSS; [51]).

As one of the main aims was to reduce the number of items in order to obtain a coherent, meaningful and quick-to-administer tool, an item reduction procedure using the Schmid-Leiman factor analysis solution [52] with maximum likelihood estimation and oblique rotation was followed for both versions (see next section for a more detailed explanation of the Schmid-Leiman factor solution and reasons why it was deemed suitable for the current data analysis task). This procedure involves recalculating the optimal factor structure after each iteration. Items were therefore screened and removed at each stage of the analysis if [a] they had high uniqueness values (>.7) (a high uniqueness value indicates that a high amount of the item’s variance is not explained by any of the factors in the model [53]), [b] they had very low loadings on either the general factor or all of the sub-factors or [c] they had similar loadings on more than one sub-factors (values between .20 and .40). Each time items were removed, a Schmid-Leiman factor model was computed again based on the optimal number of sub-factors suggested at the previous stage (this was based on the RMSEA index as well as on the interpretability of the factor solution), until all remaining items had adequate loadings on the general factor and/or on one of the sub-factors.

As a final step, a confirmatory factor analysis (CFA) procedure was employed to examine the factorial validity of the models we had constructed using the described procedure. For both the Infant and the Preschool models all factor residual variances were set to 1 and all factors were set to be uncorrelated, as suggested in [53]. The CFA was carried out using the R package lavaan [54].

## Results

### Music@Home-Preschool

First, 17 items were excluded from further analyses as they were found to have highly skewed distributions (skewness > 1.0). An initial EFA was run on the 12 dimensions that were initially hypothesized. Different criteria indicated solutions where the optimal number of factors ranged from 1 to 12. Furthermore, when performing parallel analysis the first factor yielded an eigenvalue 7 times larger than the second factor. The results above indicated the presence of a model where a general factor existed in parallel with domain-specific sub-factors [53], [55]. In other words, it was suggested that all items of the Music@Home-Preschool measure a general construct (presumably the home musical environment) while in addition facets of this construct may exist that are independent of each other after accounting for the general construct.

Our model can be specified as either a higher-order (i.e., a model where a general factor accounts for the covariance between lower-level sub-factors) or a bi-factor model (i.e., a model where a general factor exists in parallel with domain-specific sub-factors considered to be unrelated to the general factor). A bi-factor approach was deemed more appropriate in this case for two reasons: [a] In the bi-factor model a general factor accounts for the conceptual commonality between the items by having a direct influence on them, while in a higher-order model the general factor influences the items indirectly via the sub-factors [55]. In this case, the Music@Home general construct was considered to directly derive from the items while domain-specific dimensions of this construct also existed. [b] The bi-factor model is particularly useful when the researcher’s interest is to examine predictive relationships between the domain-specific dimensions of for example, a scale and other variables, after accounting for the general factor [53]. This is particularly relevant in this case as one of the aims of constructing the Music@Home instrument was to identify dimensions within this construct that could have differential effects on aspects of development.

A test using McDonald coefficient omega [56], which is a sensitive and reliable measure for the detection of a general factor [51], [57], confirmed the presence of a bi-factor structure for all factor solutions that were tested (i.e., the presence of a general factor was tested for different possible numbers of sub-factors; numbers ranged from 2 to 12). For each one of these solutions values of omega ranged from .75 to .79 (values above .6 indicate the presence of a general factor according to [56]. Furthermore, hierarchical factor analysis models that included sub-factors showed a better fit than a 1-factor model, as assessed from smaller values of the Root Mean Square Error of Approximation or RMSEA index [58].

To control for this general factor in the search for the correct number of sub-dimensions in the data, we first performed a factor analysis with maximum likelihood estimation extracting only one factor and then used the matrix of residuals for further analysis [59]. A parallel analysis on the residual matrix using Kaiser’s criterion (i.e., only factors with eigenvalues above 1 are retained) suggested the existence of 6 sub-factors while the MAP criterion suggested that four was the optimal number of factors. Both of these numbers of sub-factors were entered in the analysis to test which one gave the best model fit. The best RMSEA value (.06) was found for the model including 6 sub-factors (RMSEA index was .06, compared to .068 in the 4- sub-factor solution). This factor solution was the starting point for the item reduction procedure. To this end, the Schmid-Leiman factor solution [52] was used, which is considered suitable for bi-factor models as it calculates direct relations (these are reflected on factor loadings) between individual items and sub-factors after accounting for the impact of the general factor as well as direct relations between individual items and the general factor after accounting for the impact of sub-factors [60].

In subsequent stages, four items with high uniqueness values, 20 items that had very low loadings on either the general factor or all of the sub-factors and 7 items that had similar loadings on more than one sub-factors were removed. The Schmid-Leiman factor model was computed, until 19 items that had adequate loadings on the general and on one of the sub-factors remained. A subsequent analysis using the Schmid-Leiman procedure on those 19 items yielded a 4-factor solution with an acceptable fit to our data (RMSEA = .067) (see [58] and [61] for detailed discussions on RMSEA values that indicate an acceptable fit) and an omega value of .7. All factors had eigenvalues >1. Details of the 4 sub-factors their items and their loadings are presented in S5 Table.

Next, we assigned suitable labels to the 4 sub-factors. The 6 items that loaded on the first sub-factor included statements about musical activities (singing and music-making with either real or toy instruments) that were initiated by the parent. Therefore, the first sub-factor was labelled *Parent Initiation of Musical Behaviour*. The 4 items that loaded on the second sub-factor concerned the child’s musical activities. The second sub-factor was therefore named *Child Active Engagement with music*. The third sub-factor included 5 items and reflected parental beliefs about music and development and was therefore named *Parental Beliefs*. The fourth sub-factor included 4 items concerned with the range of musical styles that the child is exposed to within the home either via song, live instruments or electronic devices such as loudspeakers. It was therefore labelled *Breadth of Musical Exposure*.

Finally, a CFA procedure was used to test a bi-factor model that, as suggested by the EFA, was comprised of: [a] a general factor defined by all the items in the reduced version except item 19 which did not load adequately on the general factor and, [b] 4 sub-factors corresponding to the groupings above. When we ran the model on the first instance, it was observed that two items had weak loadings on their respective sub-factors (loadings < .2) indicating that these should be removed. The model was then re-run without the two items. According to the RMSEA, Bentler’s Comparative Fit Index (CFI) and Standardized Root Mean Square Residual (SRMR) fit indices the model had a good confirmatory fit to the data (see Table 1) while the Tucker-Lewis Index (TLI) was close to the cut-off of .95 [58]. Factor structure and item loadings of the Music@Home-Preschool model as formulated by CFA are presented in S1 Fig.

**Table 1:** Fit indices for the Music@Home-Preschool tested in CFA.

CFA model	χ^2^	*df*	RMSEA	CFI	TLI	SRMR
M@H-Preschool	208.49	103	.054	.945	.927	.045

Note: M@H = Music@Home, RMSEA = Root Mean Square Error of Approximation, CFI = Bentler’s Comparative Fit Index, TLI = Tucker-Lewis Index, SRMR = Standardized Root Mean Square Residual.

### Music@Home-Infant

First, 10 items were excluded from further analyses as they were found to have highly skewed distributions (> 1.2). We then proceeded to explore dimensionality of the data. As with the preschool version, different criteria suggested solutions where the optimal number of factors varied from 1 to 8. This result, as well as the fact that the first factor extracted yielded an eigenvalue five times larger than the second factor suggested the presence of a model where a general factor existed in parallel with domain-specific sub-factors [53–55]. The McDonald coefficient omega [56] value confirmed the presence of a hierarchical model for all possible numbers of sub-factors; these numbers ranged from 1 to 12. For each one of these solutions values of omega ranged from .68 to .77. Hierarchical factor analysis models that included sub-factors showed better fit than a 1-factor model.

As before we first performed a one-factor analysis and extracted the matrix of residuals. A parallel analysis on the residual matrix combined with the Kaiser’s and the MAP criterion suggested the existence of 5 sub-factors. This was taken as clear indication that five was the optimal number of sub-factors. Following the analysis of the Preschool version and since both versions were part of the same instrument we conceptualized our model as bi-factor. The Schmid-Leiman solution was therefore used to reduce the number of items and to explore the factor structure of the questionnaire.

Following the item reduction process employed with the Music@Home-Preschool, the analysis was re-run until 23 items remained. Overall, 14 items with high uniqueness values (>.7) and 13 items with either very low loadings (<.2) or similar loadings on two rather than one sub-factor (loadings between .2 and .35) were removed. A subsequent analysis on those 23 items yielded a five-factor solution (all factors had eigenvalues >1) with a very good overall fit to the data (RMSEA = .049) and a high omega value (.7). Details of the factorial structure are reported in S6 Table.

The next step was to designate appropriate labels to the 5 groupings suggested by the sub-factors. The first grouping was named *Parental beliefs* as it included items that reflected parent’s attitudes towards music and development. The second grouping of items concerned parental activities and attitudes about regulating the child’s emotion through music and singing. It was therefore labelled *Emotion Regulation*. The third grouping of items was related to the child’s engagement with musical activities and was therefore named *Child’s Active Engagement*. The fourth grouping included statements about singing activities that were initiated by the parent and was therefore labelled *Parent Initiation of Singing*. The fifth factor included items concerned with parent-child music-making and was therefore named *Parent Initiation of Music-Making*. It is important to note that analogies but also differences are observed between groupings in the Preschool and Infant version. Implications and possible interpretations of these analogies are discussed below.

Finally, a CFA procedure was employed to test a bi-factor model that, as suggested by the EFA, was comprised of: a general factor defined by all the items in the reduced version of the questionnaire and 5 sub-factors. When we ran the model in the first instance, it was observed that two of the items had weak loadings on their respective sub-factors (loadings <.2) suggesting that they should be removed. After removing these two items, the model was re-run. This model showed a good fit to the data according to the RMSEA, CFI and SRMR fit indices while the TLI index was close to the cut-off of .95 (see Table 2). Factor structure and item loadings of the Music@Home-Infant model as formulated by CFA are presented in S2 Fig.

**Table 2:** Fit indices for Music@Home -Infant tested in CFA.

CFA models	χ^2^	*df*	RMSEA	CFI	TLI	SRMR
M@H- Infant	295.32	168	.051	.947	.934	.049

Note: M@H = Music@Home, RMSEA = Root Mean Square Error of Approximation, CFI = Bentler’s Comparative Fit Index, TLI = Tucker-Lewis Index, SRMR = Standardized Root Mean Square Residual.

## Study 2: Reliability and validity of the Music@Home

The purpose of Study 2 was twofold: [a] to establish reliability and validity of both the Preschool and Infant versions of the Music@Home and, [b] to assess whether the Music@Home would be associated with musical characteristics of the parents such as active engagement with music and level of formal musical experience.

## Materials and methods

### Participants

As in Study 1, English-speaking parents of children between the ages of 3 months and 5 years and 6 months were recruited via social media, parent networks, participant databases and public mailing lists. A total of 443 individuals participated in the first instance (n = 222 for the Preschool and n = 221 for the Infant version) but 17 participants (n = 9 for the Preschool and n = 8 for the Infant version) were excluded because their children were not in the appropriate age-range.

A total of 213 participants completed the Preschool version of the Music@Home. The primary caregiver participating was in most cases the mother (*n* = 194) while in some cases it was the father (*n* = 19). The mean age of participants was 37.02 years (*SD =* 4.49). The mean age of the children for whom the survey was completed was 3.34 years (*SD* = 11.64 months). As a measure of socioeconomic status, rather than using the level of income, we used National Statistics Socio-economic Classification system (NS-SEC). The NS-SEC is the latest revised socio-economic classification recommended by the Economic and Social Research Council (ESRC) and commissioned by the Office for National Statistics (ONS) [62]. The NS-SEC derives information from occupation and employment status/size of organization to classify individuals in 5 classes; it is an internationally accepted classification and has been validated as a good predictor of educational outcomes [62]. Demographic information for participating parents and children in the preschooler sample is summarised in S7 and S8 Tables respectively.

A total of 213 participants completed the Infant version of the Music@Home. The primary caregiver participating was predominantly the mother (*n* = 206), while in few cases it was the father (*n* = 6) or other relative (*n* = 1). The mean age of participants was 35.14 years (*SD =* 4.60) while the mean age of the children was 12.81 months (*SD* = 5.71 months). Demographic information for participating parents and children in the infant sample is reported in S9 and S10 Tables respectively.

### Materials

As in Study 1, all items were entered into the *Qualtrics* online survey tool [44]. A 7-point agreement-disagreement scale was used for all items of the reduced-item versions of the Music@Home—Infant and Preschool.

With the aim of testing the convergent and divergent validity of the newly developed instrument and to examine whether there would be associations with parental characteristics relevant to musical engagement (as measured with the Gold-MSI, Goldsmiths Musical Sophistication Index, [59]) and general environmental aspects, the survey also included:

1. Two subscales from the Gold-MSI, namely the Musical Training and Active Engagement with Music subscales.
2. Five items taken from the Parent Music Activities subscale of the Children’s Music Behavior Inventory (CMBI; [42]) to test for convergent validity. These items were selected from a subset of 10 items that had the highest loadings on the Parent Music Activities factor of the CMBI (Valerio Wendy, personal communication 03/06/2016). Their selection was based on their applicability for both infants and preschoolers.
3. Two subscales from the Stim-Q Cognitive Home Environment [63] to test for divergent validity, namely the Reading and Parental Involvement in Developmental Advance scales. The Stim-Q assesses the quality of the home learning environment and similarly to the Music@Home, different versions have been developed for infants and preschoolers. Therefore, both the Stim-Q Infant and the Stim-Q Preschool were used.

### Procedure

As in Study 1, participants completed the online survey after giving their informed consent. Completion lasted approximately 20–30 minutes, provided that it was taken without pauses. As compensation for participating, parents were offered the opportunity to download five children’s songs at the end of the survey that were composed and arranged for the purposes of this research.

For the test-retest reliability phase all participants in Study 2 who had agreed to be involved in future research by providing their email addresses, received an email invitation to complete the reduced-item version of the Infant and Preschool Music@Home within 1 month of initial completion. A total of 27 participants for the Preschool version (*M*_*age of children*_ = 3.30 years, *SD* = 11.95 months) and a total of 31 participants for the Infant version (*M*_*age of infants*_ = 10.87 months, *SD* = 4.75 months) took the survey on both occasions.

### Statistical analyses

As a first step, we assessed the internal reliability of each subscale corresponding to each factor of the Music@Home questionnaires as well as of the general Music@Home factors, using three different measures, namely, Cronbach’s alpha, MacDonald’s omega total, and Guttman’s lambda 6. Scores for each Music@Home subscale and for the general factors were calculated by summing item scores.

To establish factorial validity of the Music@Home, we employed confirmatory factor analysis (CFA) using the R package lavaan [54] to test whether the models constructed in Study 1 using the Schmid-Leiman solution would fit the data from the sample collected in Study 2. Therefore, for both Preschool and Infant versions, a bi-factor model was tested where the general factor impacted directly on all items (i.e., all items loading directly on the general factor) while the sub-factors also impacted on the items associated with them (i.e., individual items also loaded on their respective factors). It is important to note that item 13 of the Music@Home-Preschool (see S5 Table) was only included in the specification of the Parent Initiation of Musical Behaviour subscale, as its loading to the general factor in Study 1 was low. Finally, we performed correlational analyses to assess convergent and divergent validity of our instrument and to examine associations between the Music@Home (general factor and subscales) and two subscales from the Gold-MSI, i.e., Musical Training and Active Engagement with Music subscales.

## Results

As can be seen in Table 3, all subscales of the Preschool version showed moderate to very good reliability estimates. Similarly, all subscales of the Infant version showed moderate to very good reliability apart from Emotion Regulation. Table 3 also presents test-retest reliability correlations. All subscales in both versions, apart from the Emotion regulation subscale of the Music@Home-Infant, displayed high test-retest correlations (.647 to .871) significant at the *p* < .001 level. The test-retest correlation for the Emotion Regulation subscale was non-significant.

**Table 3:** Estimates of internal reliability and test-retest reliability correlations for the Music@Home-Preschool and Music@Home-Infant factors.

	alpha	omega.tot	G6	test-retest
**M@H-Preschool general factor**	.851	.871	. 895	.83***
Parental beliefs	.710	.735	. 674	.87***
Child’s active engagement	.765	.781	. 746	.68***
Parent initiation of musical behaviour	.799	.804	. 766	.82***
Breadth of musical exposure	.662	.676	. 602	.81***
**M@H-Infant general factor**	.873	.879	.909	.69***
Parental beliefs	.693	.717	.637	.82***
Emotion regulation	.569	.584	.480	.21
Child’s active engagement	.835	.842	.832	.67***
Parent initiation of singing	.808	.820	.793	.64***
Parent initiation of music-making	.857	.861	.803	.68***

Note:

***p <.001

Note: The final version of the Music@Home-Infant did not include the Emotion Regulation subscale.

Note: Reliability measures for the Music@Home-Infant general factor before the Emotion Regulation subscale was removed: internal reliability: alpha = .876, omega tot = .883, lambda G6 = .911, test-retest reliability: *r* = .657, *p* < .001.

Given that these results suggested that the correspondence of the Emotion Regulation items to a separate grouping is questionable, this subscale and its items were excluded from further analyses. Reliability of the Music@Home –Infant general factor was then re-calculated. This analysis returned very good internal and test-retest reliability (see Table 3).

**Fig 1:**
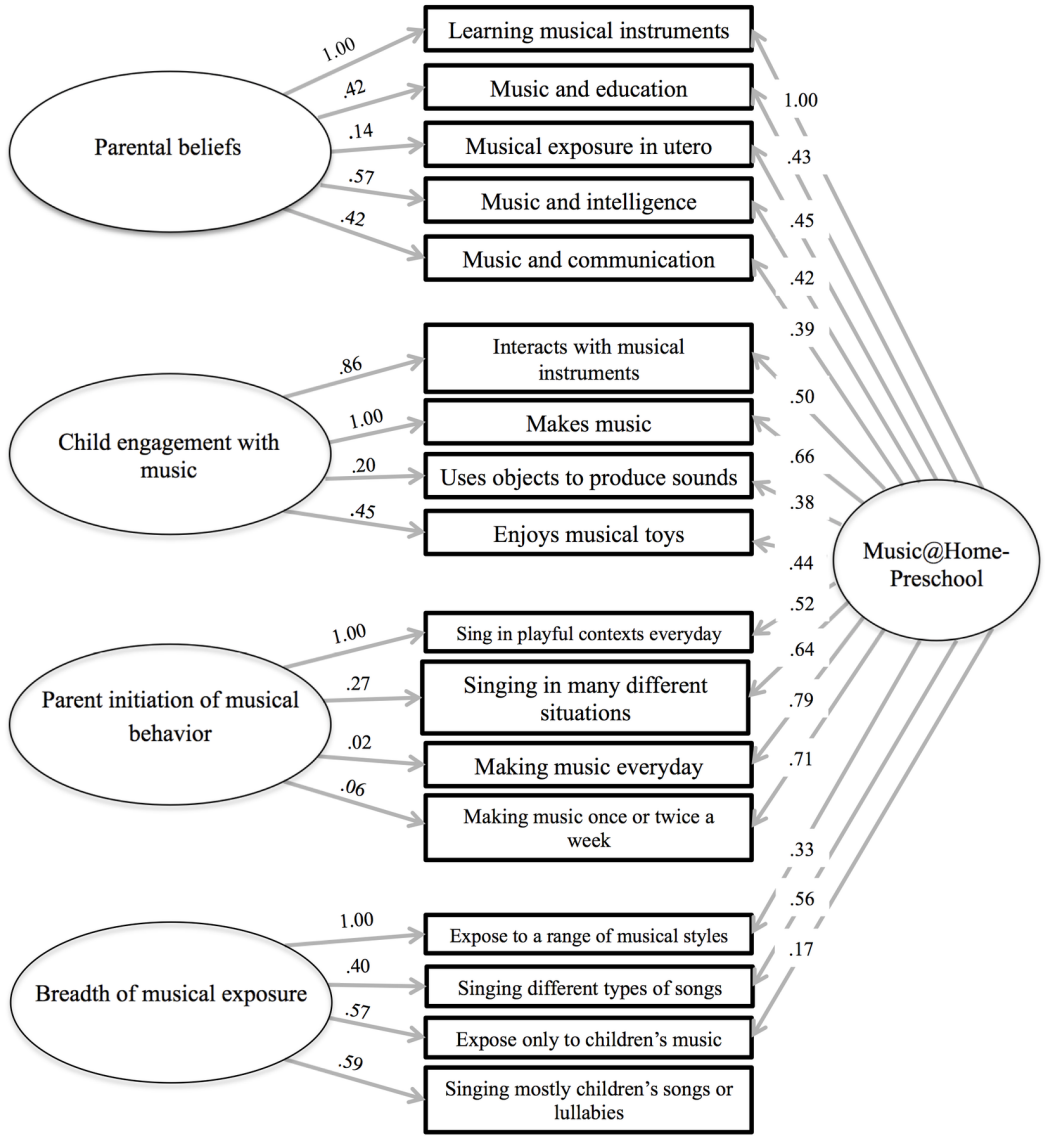
Factor structure of the Music@Home-Preschool as formalized by confirmatory factor analysis.

**Fig 2:**
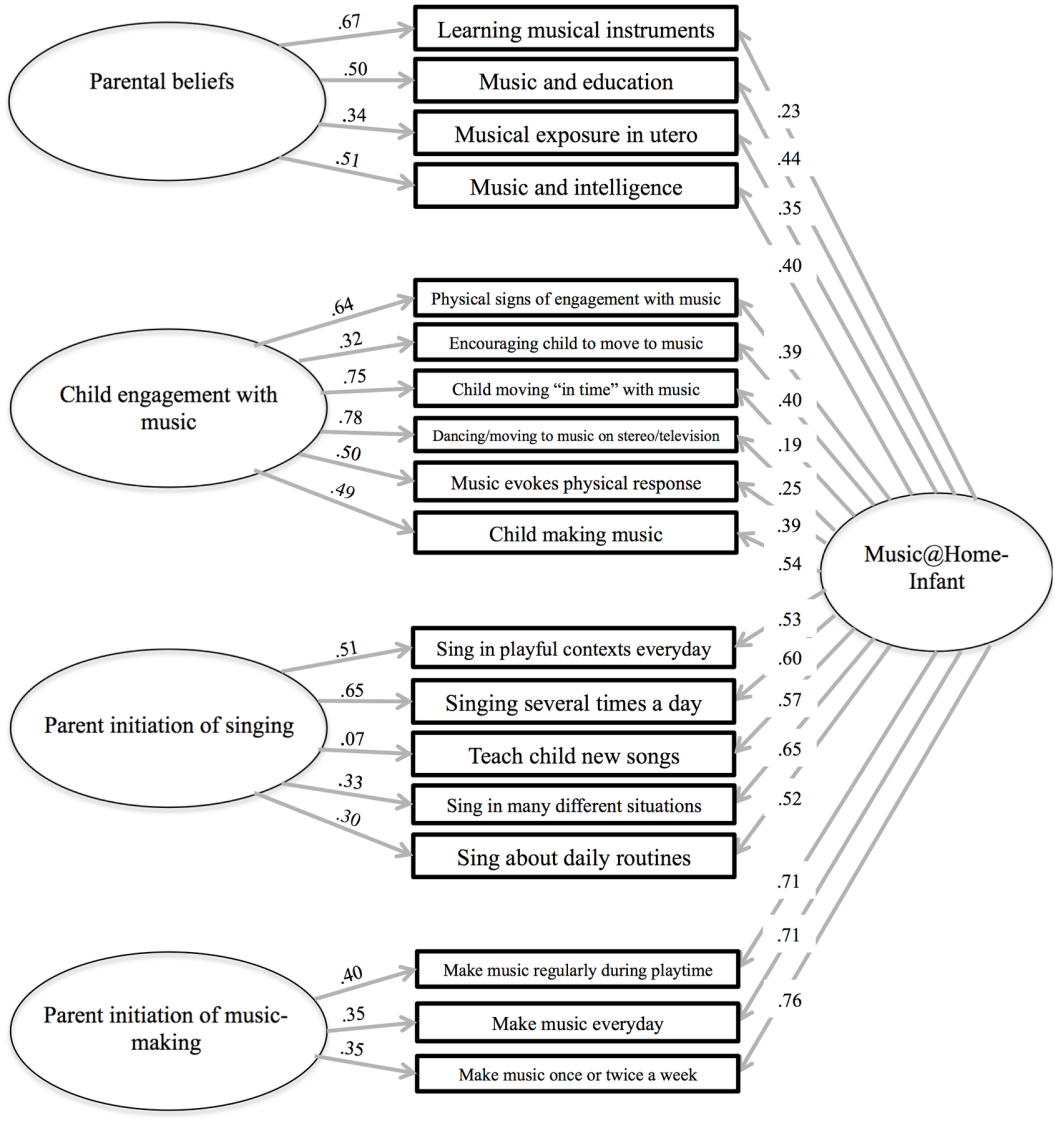
Factor structure of the Music@Home-Infant as formalized by confirmatory factor analysis.

Confirmatory fit indices for both versions of the Music@Home questionnaire are presented in Table 4. Fit indices for the Music@Home-Preschool model are within the acceptable range while the Music@Home-Infant shows very good confirmatory fit to the data. Factor structure and item loadings of both CFA models are presented in Figs 1 and 2.

**Table 4:** Fit indices for the Music@Home-Preschool and Infant models assessed with CFA.

Models	χ^2^	df	RMSEA	CFI	TLI	SRMR
M@H-Preschool	222.055	105	.072	.872	.834	.070
M@H-Infant	161.761	117	.042	.963	.952	.049

Note: M@H = Music@Home, RMSEA = Root Mean Square Error of Approximation, CFI = Bentler’s Comparative Fit Index, TLI = Tucker-Lewis Index, SRMR = Standardized Root Mean Square Residual.

As shown in Table 5, both versions of the Music@Home and their associated subscales show highly significant correlations with the CMBI, establishing convergent validity of the scale. Furthermore, both the Preschool and Infant versions of our instrument exhibit low to moderate associations with the Reading and PIDA subscales of the Stim-Q, suggesting that the Music@Home measures a unique form of engagement that can be disentangled from general engagement of parents with their children. Interestingly, the Child Engagement with Music subscale of the Music@Home-Infant shows high correlations with both the Reading and PIDA subscales. As it is unlikely that this subscale, which refers to the child’s behaviour, measures general parental engagement, this result might indicate a connection between the quantity and quality of parent-child engagement in various activities and the child’s attitude towards these activities. Moderate but significant correlations are observed between the majority of the Music@Home Infant subscales and both Active Engagement and Musical Training scores for parents’ from the Gold-MSI. In contrast, the Music@Home Preschool factors show significant correlations with Active Engagement but not with Musical Training.

**Table 5:** Convergent and divergent validity of the Music@Home Preschool and Infant versions and associations with parents’ musical background (Musical Training and Active Engagement subscales from the Gold-MSI) as well as the quality of the home learning environment (reading and parental involvement in developmental advance subscales from the StimQ inventory).

	CMBI	StimQ-Reading	StimQ-PIDA	Gold-MSI -Act Eng	Gold-MSI—MusTrai
M@H-Preschool general factor	.49***	.09	.04	.40***	.05
Parental beliefs	.45***	.11	.03	.39***	.05
Child’s active engagement	.37***	-.02	-.02	.17*	.03
Parent init of mus behaviour	.46***	.07	.03	.36***	.05
Breadth of musical exposure	.24***	.13	.08	.34***	-.00
M@H-Infant general factor	.53***	.12	.20**	.24***	.23***
Parental beliefs	.40***	-.11	.03	.38***	.37***
Child’s active engagement	.32***	.27***	.33***	.07	.06
Parent init of singing	.38***	.03	.08	.18*	.14*
Parent init of music-making	.48***	.06	.07	.14*	.17*

Note:

*p <.05,

**p <.01,

***p <.001,

M@H = Music@Home, Parent init = Parent initiation, CMBI = Children’s Music Behavior Inventory, PIDA = Parental Involvement in Developmental Advance, Gold-MSI-MusTrai = Gold-MSI-Musical Training.

## Discussion

The purpose of the present study was to develop and evaluate a comprehensive and systematic parent-report instrument, capable of assessing the musical home environment for children under the age of 5. Given qualitative differences in the way parents may engage musically with infants relative to preschoolers, two versions of the new Music@Home were developed, namely, Preschool and Infant.

Results from Study 1 showed that the Music@Home experience for both infants and preschoolers was best described as comprising a general factor corresponding to the home musical environment, while dimensions or sub-factors of this construct existed in parallel [53–55]. These sub-factors quantify a range of dimensions relevant to the informal musical environment in the home. The factor structures of the Infant and the Preschool versions differed in terms of sub-factors they included, reflecting variations in the musical engagement and nature of parent-child interactions between the two age groups. The dimensions identified and the parallels and differences between the Infant and the Preschool versions are outlined below, and provide new intuitions about early musical experience in the home adding to previous research that has explored this concept either for descriptive purposes [28–33] or in music educational contexts [32], [41–43].

With respect to the Music@Home-Preschool, four factors were identified. These corresponded to the following dimensions: Parental Beliefs, Child Engagement, Breadth of Musical Exposure and Parent Initiation of Musical Behaviour. A variant of this structure was identified for the Infant version, the factors of which were assigned the following names: Parental Beliefs, Child Engagement, Emotion Regulation, Parent Initiation of Singing, and Parent Initiation of Music-making. Notably, the Emotion Regulation subscale exhibited low internal and test-retest reliability and was subsequently discarded.

“Parental beliefs” represents notions of the parents with regards to the beneficial effects of music to their child’s general development. Importantly, the same cluster of questions that reflected parental beliefs in the Music@Home—Preschool were also uncovered during the -Infant version analysis, suggesting that this is a facet of the home musical environment possibly independent of a child’s age. The Preschool version however, included an extra item (“I think musical activities are important for learning to communicate”) reflecting the expanded communicative repertoire of preschoolers. This aspect has been previously addressed in studies looking to describe the musical home environment of infants and young children [28], [30–31], [43], [64–65] or exploring music-related parental attitudes in relation to music educational outcomes [41], [66–69]. These studies have shown that parents’ whose children participate in music classes usually have positive beliefs towards the general educational benefits and significance of music practice [66], [68], and that parental attitudes can be positively associated with children’s musical attainment and motivation [41], [67], [69]. Furthermore, the majority of parents of preschoolers who expressed positive beliefs about the benefits of music education also reported high frequency of singing and listening to music with their children [31]. Qualitative analyses of parental responses to surveys examining musical engagement in the home, have also provided illustrative examples of how parents’ attitudes and beliefs about the benefits and functions of music influence the use of music in the home environments of infants [30] preschoolers [64] and young children [65]. Taken together, the above findings indicate that parental attitudes and beliefs are important influences on children’s musical experience in formal or informal settings. Therefore, this subscale provides a consistent measurement of a dimension that will contribute to providing a complete picture of the home musical environment.

The “Child engagement” subscale, which represents children’s active participation and initiation of musical activities, was a factor that surfaced in both infant and preschool versions of the Music@Home. Musical behaviours that emerged as important for each version reflected characteristics of the different age groups (e.g., example item from the infant version: “Music does not evoke a physical response from my child”; example item from the preschool version: “My child enjoys making sounds/interacting with musical instruments, including toy ones”). Children’s musical engagement and participation has been thoroughly addressed in the work of Valerio et al. [42] who constructed a parent-report questionnaire with the specific aim of documenting preschool children’s musical behaviour (the Children’s Musical Behavior Inventory or CMBI) in order to best meet the musical needs in childcare and school settings. Although this is the only existing validated measurement of children’s musical engagement, the importance of observing and documenting aspects of the child’s music-related behaviour in musical development research and music education has long been recognized [43], [70–75]. Including a relevant subscale in a systematic instrument assessing informal musical home environment such as the Music@Home opens new avenues for exploring, in experimental contexts, how the interplay of parent-child characteristics may affect the child’s experience as well as children’s developmental outcomes.

The “Parent Initiation of Musical Behaviour” subscale of the Music@Home-Preschool indexes parent-triggered musical engagement, such as singing and making music with the child. Undoubtedly, parent-child musical interactions are a crucial aspect of the home musical environment and a higher frequency of these activities during the early years has previously been associated with positive outcomes, such as enhanced auditory sensitivity [37] and better vocabulary skills [38]. Along with parental beliefs and attitudes, parental musical involvement with their children at home, has been recognized as an important influence in children’s musical attainment [41].

Although in the Music@Home-Preschool singing and music making comprised a single factor exhibiting very good internal reliability, these two activities emerged as separate factors in the Music@Home-Infant. The distinction between the two versions’ factor structure presumably reflects differences in the extent to which parents of different age groups engage in the two activities. Clearly, infants until the age of 2 years engage in active music—making to a lesser extent, while parental singing appears to hold a central role in regulating arousal [21] and building emotional interaction [76–77] in infancy. Furthermore, approaching singing in infancy as a separate dimension highlights the importance that this activity may carry for developmental outcomes such as socio-emotional and communicative development [78] and language learning [24– 25]. Indeed, due to the rhythmic and melodic properties of song that emphasize and exaggerate speech elements, singing has been shown to facilitate phonetic learning in 6- to 8- and in 11-month-olds [24–25]. Another possibility is that infants benefit from the combined input of music and lyrics, since a second source of information (music) provides additional cues to help them identify structure in the first source (words and syllables) [25]. Furthermore, Van Puyvelde and Franco [78] have proposed that the melodic patterns and moments of ‘tonal synchrony’ observed in parent-infant vocal interactions [79], which facilitate affective co-regulation [77] may well be a prerequisite for later social development. Therefore, since one of the key aims of developing the Music@Home questionnaire is its future use in experimental research, addressing the potential effects that aspects of the home musical environment may have on development, including parental singing as a separate subscale for the infant version is well motivated and supported by existing literature.

Another dimension that emerged in the Music@Home-Preschool but not the Infant version refers to the breadth of musical exposure in the home, including music that is sung or listened to. This grouping of items reveals an aspect of the musical home environment that has not previously been addressed as a separate dimension of potential importance. Questionnaires or interviews in previous studies have embedded items about the quantity of music heard in the home or the number of musical resources such as CD’s, musical and toy instruments [28], [33], [40–41]. However, exposing the child to a broad range of musical styles either via song or music listening may reflect qualitative differences in individuals’ appreciation of music and motivation to include their children in their musical interests. Not surprisingly, this factor was associated with personal engagement of parents with music. However, it was not correlated with musical training suggesting that it does not represent formal musical experience but potentially reflects a unique type of musical sophistication that may extend to how parents interact musically with their children. Exploring the associations of this dimension with other potential variables such as personality characteristics is an interesting question for future studies.

It is important to note that one of the factors that was initially revealed as relevant to the Music@Home-Infant experience, namely “Emotion Regulation”, showed low reliability when evaluated with a new sample of participants. This is surprising, given that mood regulation appears to be a central function of music and singing in the infant years [33], [35], [70]. One possibility is that a very small number of items were included in this subscale contributing to low reliability of the factor. Another possibility is that the negative items in the subscale (two of three items were negatively worded) were unclear to participants leading them to respond erratically thus affecting intercorrelations between items and compromising internal reliability. As test-retest reliability of this subscale was also low, it was subsequently removed from further analysis.

Strong associations were observed between all subscales of the Music@Home-Preschool and Infant, and a subscale of the CMBI measuring parental involvement with music (i.e., Parent Music Activities). This establishes convergent validity of the Music@Home questionnaires. Notably, although highly significant, correlation coefficients were not higher than .6, suggesting that Music@Home measures a relevant but not identical construct to the CMBI-Parent Music Activities. Divergent validity of the questionnaires was also established, as weak to moderate associations were found between most of the dimensions of the Music@Home Preschool and Infant, and two subscales from a validated instrument (i.e. Stim-Q) assessing the learning environment of young children at home (Reading and Parental Involvement to Developmental Advance or PIDA). These results suggest that the music at home experience as assessed with our newly developed questionnaire can be disentangled from general engagement of parents with their children. A moderately significant correlation that was observed between the Music@Home-Infant general factor and the PIDA subscale suggests that in the case of infants, there is a stronger coupling between engaging in musical activities and promoting learning in other domains.

Interestingly, stronger associations were observed between the Child Engagement subscale of the Music@Home-Infant and both the Reading and PIDA subscales. However, since this subscale reflects infant attitudes and behaviours towards music, it is unlikely that it assesses a concept similar to general parental engagement. Rather, it is possible that this finding hints at an association between the level of parent-child engagement in various activities and the child’s attitude towards these activities. In line with this view Kuhl [80–81] has argued that interpersonal engagement can have determinant effects on infant attention and arousal.

Further supporting the above argument, Child Engagement from the infant version was the only factor that was not related to parental musical characteristics as measured with the Gold-MSI subscales (Active Engagement and Musical Training). This suggests that, on one hand, young infants are not yet in a position to imitate or be influenced by their parents’ personal interests and on the other hand, that they may benefit more from active participation in joint activities.

Differential associations emerged between the subscales of the Gold-MSI and the two versions of the Music@Home. More specifically, the general factor and all subscales of the Music@Home –Preschool showed strong associations with parent’s personal engagement with music (Active Engagement factor) but not with their level of formal musical training (Musical Training factor*)*. On the other hand, the Music@Home-Infant general factor and most of the subscales showed strong associations with both these factors, consistently with Custodero and Johnson-Green [43] who found that musically trained parents were more likely to engage musically with their infants. These findings possibly reflect differences between the two age groups’ characteristics and modes of interaction. In the case of preschoolers, where there is a more limited time window for parent-child engagement and a broadening of external influences due to changes in everyday activities (e.g., parents may be back at work, children may be attending nursery), it is the parents’ level of personal engagement with music that affects the mode of interactions with their children, whereas musical training (which may have been received some years ago) does not determine their current level of joint activities to the same extent. In the case of infants however, there might be a greater opportunity for parental characteristics to be reflected in the way they interact with their young ones, either due to spending a greater amount of time at home with infants relative to preschoolers (see [82] for statistics on children attending day care in function of age), or because personal inclinations may weigh more in orienting the choice of activities at developmental stages in which children have more limited vocal and motor abilities. Future studies need to address these alternatives more specifically in order to elucidate their potential role in shaping everyday musical experiences at the start of the developmental path.

In conclusion, the Music@Home Preschool and Infant questionnaires provide researchers and educators with a novel, quick-to-administer, valid and reliable instrument for the systematic assessment of the home musical environment. Importantly, our instrument brings together for the first time novel dimensions such as breadth of musical exposure, and combines them with more typically studied aspects of musical experience, allowing for a comprehensive tool to capture the extent of musical activities occurring informally within the home. Future studies can make use of this systematic instrument to explore the nature of the home musical environment and examine its associations with individual and parent characteristics. Crucially, we envisage this questionnaire to be of use in research investigating the potential contribution of the home musical environment to various developmental outcomes.

## Supporting information

S1 Table Study 1: Music@Home-Preschool: Demographic information for participating parents. (DOCX)

S2 Table Study 1: Music@Home-Preschool: Demographic information for the respondents’ children. (DOCX)

S3 Table Study 1: Music@Home-Infant: Demographic information for participating parents. (DOCX)

S4 Table Study 1: Music@Home-Infant: Demographic information for the respondents’ children. (DOCX)

S5 Table Study 1: Structure of factors and item loadings for the Music@Home—Preschool. (DOCX)

S6 Table Study 1: Structure of factors and item loadings for the Music@Home—Infant. (DOCX)

S7 Table Study 2: Music@Home-Preschool: Demographic information for participating parents. (DOCX)

S8 Table Study 2: Music@Home-Preschool: Demographic information for the respondents’ children. (DOCX)

S9 Table Study 2: Demographic information for Music@Home-Infant participating parents. (DOCX)

S10 Table Study 2: Music@Home-Infant: Demographic information for the respondents’ children. (DOCX)

S1 Fig Study 1: Factor structure of the Music@Home-Preschool as formalized by confirmatory factor analysis. (PDF)

S2 Fig Study 1: Factor structure of the Music@Home-Infant as formalized by confirmatory factor analysis. (PDF)

## References

[1] Moreno S, Bialystok E, Barac R, Schellenberg EG, Cepeda NJ, Chau T. Short-term music training enhances verbal intelligence and executive function. Psychological science. 2011;22(11): 1425–1433. doi: 10.1177/0956797611416999 21969312 10.1177/0956797611416999 PMC3449320

[2] Degé F, Schwarzer G. The effect of a music program on phonological awareness in preschoolers. Frontiers in psychology. 2011;2. 10.3389/fpsyg.2011.00124 PMC3121007 21734895

[3] François C, Chobert J, Besson M, Schön D. Music training for the development of speech segmentation. Cerebral Cortex. 2012;23(9): 2038–2043. doi: 10.1093/cercor/bhs180 22784606 10.1093/cercor/bhs180

[4] Flohr JW. Musical lives of young children. New Jersey: Prentice Hall; 2005.

[5] Hoff E. The specificity of environmental influence: Socioeconomic status affects early vocabulary development via maternal speech. Child development. 2003;74(5): 1368–1378. 14552403 10.1111/1467-8624.00612

[6] Huttenlocher J, Waterfall H, Vasilyeva M, Vevea J, Hedges LV. Sources of variability in children’s language growth. Cognitive psychology. 2010;61(4): 343–365. doi: 10.1016/j.cogpsych.2010.08.002 20832781 10.1016/j.cogpsych.2010.08.002 PMC2981670

[7] Pan BA, Rowe ML, Singer JD, Snow CE. Maternal correlates of growth in toddler vocabulary production in low‐income families. Child development. 2005;76(4): 763–782. 16026495 10.1111/j.1467-8624.2005.00876.x

[8] Rowe ML, Goldin-Meadow S. Differences in early gesture explain SES disparities in child vocabulary size at school entry. Science. 2009;323(5916): 951–953. doi: 10.1126/science.1167025 19213922 10.1126/science.1167025 PMC2692106

[9] Weisleder A, Fernald A. Talking to children matters: Early language experience strengthens processing and builds vocabulary. Psychological science. 2013;24(11): 2143–2152. doi: 10.1177/0956797613488145 24022649 10.1177/0956797613488145 PMC5510534

[10] Sénéchal M, Pagan S, Lever R, Ouellette GP. Relations among the frequency of shared reading and 4-year-old children’s vocabulary, morphological and syntax comprehension, and narrative skills. Early Education and Development. 2008;19(1): 27–44.

[11] Anders Y, Rossbach HG, Weinert S, Ebert S, Kuger S, Lehrl S, von Maurice J. Home and preschool learning environments and their relations to the development of early numeracy skills. Early Childhood Research Quarterly. 2012;27(2): 231–244.

[12] Kleemans T, Peeters M, Segers E, Verhoeven L. Child and home predictors of early numeracy skills in kindergarten. Early Childhood Research Quarterly. 2012;27(3): 471–477.

[13] Baker CE, Cameron CE, Rimm-Kaufman SE, Grissmer D. Family and sociodemographic predictors of school readiness among African American boys in kindergarten. Early Education & Development. 2012;23(6): 833–854.

[14] Chazan-Cohen R, Raikes H, Brooks-Gunn J, Ayoub C, Pan BA, Kisker EE, Roggman L, Fuligni AS. Low-income children’s school readiness: Parent contributions over the first five years. Early Education and Development. 2009;20(6): 958–977.

[15] Hartas D. Families’ social backgrounds matter: Socio-economic factors, home learning and young children’s language, literacy and social outcomes. British Educational Research Journal. 2011;37(6): 893–914.

[16] Fernald A, Kuhl P. Acoustic determinants of infant preference for motherese speech. Infant behavior and development. 1987;10(3): 279–293.

[17] Fernald A, Taeschner T, Dunn J, Papousek M, de Boysson-Bardies B, Fukui I. A cross-language study of prosodic modifications in mothers’ and fathers’ speech to preverbal infants. Journal of child language. 1989;16(3): 477–501. 2808569 10.1017/s0305000900010679

[18] Trehub SE, Unyk AM, Kamenetsky SB, Hill DS, Trainor LJ, Henderson JL, Saraza M. Mothers’ and fathers’ singing to infants. Developmental psychology. 1997;33(3): 500 9149928 10.1037//0012-1649.33.3.500

[19] Trehub SE, Unyk AM, Trainor LJ. Maternal singing in cross-cultural perspective. Infant behavior and development. 1993;16(3): 285–295.

[20] Trehub SE, Hill DS, Kamenetsky SB. Parents’ sung performances for infants. Canadian Journal of Experimental Psychology/Revue canadienne de psychologie expérimentale. 1997;51(4): 385 9606951 10.1037/1196-1961.51.4.385

[21] Shenfield T, Trehub SE, Nakata T. Maternal singing modulates infant arousal. Psychology of Music. 2003;31(4): 365–375.

[22] Trehub SE, Plantinga J, Russo FA. Maternal vocal interactions with infants: Reciprocal visual influences. Social Development. 2016; 25(3): 665–683.

[23] Costa-Giomi E. Mode of presentation affects infants’ preferential attention to singing and speech. Music Perception: An Interdisciplinary Journal. 2014;32(2): 160–169.

[24] Lebedeva GC, Kuhl PK. Sing that tune: Infants’ perception of melody and lyrics and the facilitation of phonetic recognition in songs. Infant behavior and development. 2010;33(4): 419–430. doi: 10.1016/j.infbeh.2010.04.006 20472295 10.1016/j.infbeh.2010.04.006 PMC2943554

[25] Thiessen ED, Saffran JR. How the melody facilitates the message and vice versa in infant learning and memory. Annals of the New York Academy of Sciences. 2009;1169(1): 225–233. 19673786 10.1111/j.1749-6632.2009.04547.x

[26] Schön D, Boyer M, Moreno S, Besson M, Peretz I, Kolinsky R. Songs as an aid for language acquisition. Cognition. 2008;106(2): 975–983. doi: 10.1016/j.cognition.2007.03.005 17475231 10.1016/j.cognition.2007.03.005

[27] Costa-Giomi E, Ilari B. Infants’ preferential attention to sung and spoken stimuli. Journal of Research in Music Education. 2014;62(2): 188–194.

[28] Ilari B. On musical parenting of young children: Musical beliefs and behaviors of mothers and infants. Early Child Development and Care. 2005;175(7–8): 647–660.

[29] Shoemark H, Arnup S. A survey of how mothers think about and use voice with their hospitalized newborn infant. Journal of Neonatal Nursing. 2014;20(3): 115–121.

[30] De Vries P. Music at home with the under fives: What is happening?. Early Child Development and Care. 2009;179(4): 395–405.

[31] Mehr SA. Music in the home: New evidence for an intergenerational link. Journal of Research in Music Education. 2014;62(1): 78–88.

[32] Youm HK. Parents’ goals, knowledge, practices, and needs regarding music education for their young children in South Korea. Journal of Research in Music Education. 2013;61(3): 280–302.

[33] Young S. Lullaby light shows: Everyday musical experience among under-two-year-olds. International Journal of Music Education. 2008;26(1): 33–46.

[34] Barrett MS. Musical narratives: A study of a young child’s identity work in and through music-making. Psychology of Music. 2011;39(4): 403–423.

[35] Barrett MS. Sounding lives in and through music: a narrative inquiry of the everyday musical engagement of a young child. Journal of Early Childhood Research. 2009;7(2): 115–134.

[36] Ilari B, Young S. Children’s home musical experiences across the world. Indiana: Indiana University Press; 2016.

[37] Putkinen V, Tervaniemi M, Huotilainen M. Informal musical activities are linked to auditory discrimination and attention in 2–3-year-old children: an event-related potential study. European Journal of Neuroscience. 2013;37(4): 654–661. doi: 10.1111/ejn.12049 23167769 10.1111/ejn.12049

[38] Williams KE, Barrett MS, Welch GF, Abad V, Broughton M. Associations between early shared music activities in the home and later child outcomes: Findings from the Longitudinal Study of Australian Children. Early Childhood Research Quarterly. 2015;31: 113–124.

[39] Farrant BM, Zubrick SR. Parent–child book reading across early childhood and child vocabulary in the early school years: Findings from the Longitudinal Study of Australian Children. First Language. 2013;33(3): 280–293.

[40] Brand M. Development and validation of the home musical environment scale for use at the early elementary level. Psychology of Music. 1985;13(1): 40–48.

[41] Brand M. Relationship between home musical environment and selected musical attributes of second-grade children. Journal of Research in Music Education. 1986;34(2): 111–120.

[42] Valerio WH, Reynolds AM, Morgan GB, McNair AA. Construct validity of the children’s music-related behavior questionnaire. Journal of Research in Music Education. 2012;60(2): 186–200.

[43] Custodero LA, Johnson-Green EA. Passing the cultural torch: Musical experience and musical parenting of infants. Journal of Research in Music Education. 2003;51(2): 102–114.

[44] Qualtrics. Provo: Utah; 2017. http://www.qualtrics.com [Accessed 13th July 2015].

[45] R Core Team: A language and environment for statistical computing. R Foundation for Statistical Computing Vienna: Austria; 2012. http://www.R-project.org/ [Accessed 10^th^ January 2015].

[46] Revelle WR. psych: Procedures for personality and psychological research. Northwestern University, Evanston, Illinois, USA. 2017, https://CRAN.R-project.org/package=psych Version=1.7.8.

[47] Horn JL. A rationale and test for the number of factors in factor analysis. Psychometrika. 1965 6 27;30(2):179–85. 14306381 10.1007/BF02289447

[48] Dinno A. Implementing Horn’s parallel analysis for principal component analysis and factor analysis. Stata Journal. 2009;9(2): 291.

[49] Cattell RB. The scree test for the number of factors. Multivariate behavioral research. 1966;1(2): 245–276. doi: 10.1207/s15327906mbr0102_10 26828106 10.1207/s15327906mbr0102_10

[50] Velicer WF. Determining the number of components from the matrix of partial correlations. Psychometrika. 1976;41(3): 321–327.

[51] Revelle W, Rocklin T. Very simple structure: An alternative procedure for estimating the optimal number of interpretable factors. Multivariate Behavioral Research. 1979; 14(4):403–414. doi: 10.1207/s15327906mbr1404_2 26804437 10.1207/s15327906mbr1404_2

[52] Schmid J, Leiman JM. The development of hierarchical factor solutions. Psychometrika. 1957;22(1): 53–61.

[53] Chen FF, West SG, Sousa KH. A comparison of bifactor and second-order models of quality of life. Multivariate Behavioral Research. 2006;41(2): 189–225. 26782910 10.1207/s15327906mbr4102_5

[54] Oberski DL. lavaan. survey: An R package for complex survey analysis of structural equation models. Journal of Statistical Software. 2014 3 13;57(1):1–27.25400517

[55] Beaujean AA. Latent variable modeling using R: A step-by-step guide. New York: Routledge; 2014.

[56] McDonald RP. Test theory: A unified treatment. New Jersey: Erlbaum; 1999.

[57] Revelle W, Wilt J. The general factor of personality: A general critique. Journal of research in personality. 2013;47(5): 493–504. doi: 10.1016/j.jrp.2013.04.012 23956474 10.1016/j.jrp.2013.04.012 PMC3743124

[58] Hu LT, Bentler PM. Cutoff criteria for fit indexes in covariance structure analysis: Conventional criteria versus new alternatives. Structural equation modeling: a multidisciplinary journal. 1999;6(1): 1–55.

[59] Müllensiefen D, Gingras B, Musil J, Stewart L. The musicality of non-musicians: an index for assessing musical sophistication in the general population. PloS one. 2014;9(2): e89642 doi: 10.1371/journal.pone.0089642 24586929 10.1371/journal.pone.0089642 PMC3935919

[60] Wolff HG, Preising K. Exploring item and higher order factor structure with the Schmid-Leiman solution: Syntax codes for SPSS and SAS. Behavior Research Methods. 2005 2 1;37(1):48–58. 16097343 10.3758/bf03206397

[61] MacCallum RC, Browne MW, Sugawara HM. Power analysis and determination of sample size for covariance structure modeling. Psychological methods. 1996;1(2): 130.

[62] Rose D, Pevalin DJ, O’Reilly K. The National Statistics Socio-economic Classification: origins, development and use. Basingstoke: Palgrave Macmillan; 2005.

[63] Dreyer BP, Mendelsohn AL, Tamis-LeMonda CS. Stim-Q Cognitive Home Environment. https://med.nyu.edu/pediatrics/developmental/research/belle-project/stimq-cognitive-home-environment [Accessed on 24th January 2016].

[64] Custodero LA, Johnson-Green EA. Caregiving in counterpoint: Reciprocal influences in the musical parenting of younger and older infants. Early Child Development and Care. 2008 1 1;178(1):15–39.

[65] Ilari B. Middle-class musical childhoods In: Ilari B, Young S, editors. Children’s home musical experiences across the world. Indiana: Indiana University Press; 2016 p. 92–105.

[66] Cho E. What do mothers say? Korean mothers’ perceptions of children’s participation in extra-curricular musical activities. Music Education Research. 2015;17(2): 162–178.

[67] Driscoll V, Gfeller K, Tan X, See RL, Cheng HY, Kanemitsu M. Family involvement in music impacts participation of children with cochlear implants in music education and music activities. Cochlear implants international. 2015;16(3): 137–146. doi: 10.1179/1754762814Y.0000000103 25431978 10.1179/1754762814Y.0000000103 PMC4420640

[68] Dai DY, Schader RM. Decisions regarding music training: Parental beliefs and values. Gifted Child Quarterly. 2002;46(2): 135–144.

[69] Sichivitsa VO. The influences of parents, teachers, peers and other factors on students’ motivation in music. Research Studies in Music Education. 2007;29(1): 55–68.

[70] Custodero LA. Singing practices in 10 families with young children. Journal of Research in Music Education. 2006;54(1): 37–56.

[71] Custodero LA, Britto PR, Brooks-Gunn J. Musical lives: A collective portrait of American parents and their young children. Journal of Applied Developmental Psychology. 2003;24(5): 553–572.

[72] Moog H. The development of musical experience in children of pre-school age. Psychology of Music. 1976 10;4(2):38–45.

[73] Rainbow E. A final report on a three-year investigation of the rhythmic abilities of preschool aged children. Bulletin of the Council for Research in Music Education. 1981:69–73.

[74] Rutkowski J. The nature of children’s singing voices: Characteristics and assessment In: Roberts BA, editor. The phenomenon of singing. NewFoundland/Labrador: Memorial University Press; 2013 pp. 201–209.

[75] Welch GF. Singing and vocal development In: McPherson G, editor. The child as musician: A handbook of musical development. Oxford: Oxford University Press; 2006 pp. 311–329.

[76] Nakata T, Trehub SE. Infants’ responsiveness to maternal speech and singing. Infant Behavior and Development. 2004;27(4): 455–464.

[77] Van Puyvelde M, Loots G, Vanfleteren P, Meys J, Simcock D, Pattyn N. Do You Hear the Same? Cardiorespiratory responses between mothers and infants during tonal and atonal music. PloS one. 2014;9(9): e106920 doi: 10.1371/journal.pone.0106920 25207803 10.1371/journal.pone.0106920 PMC4160208

[78] Van Puyvelde M, Franco, F. ‘The interaction of music and language in the ontogenesis of human communication: A multimodal parent-infant co-regulation system’. http://hridev1.shef.ac.uk/openbook/chapter/ICMEM_39 [Accessed 18th August 2015].

[79] Van Puyvelde M, Vanfleteren P, Loots G, Deschuyffeleer S, Vinck B, Jacquet W, Verhelst W. Tonal synchrony in mother–infant interaction based on harmonic and pentatonic series. Infant behavior and development. 2010;33(4): 387–400. doi: 10.1016/j.infbeh.2010.04.003 20478620 10.1016/j.infbeh.2010.04.003

[80] Kuhl PK, Tsao FM, Liu HM. Foreign-language experience in infancy: Effects of short-term exposure and social interaction on phonetic learning. Proceedings of the National Academy of Sciences. 2003;100(15): 9096–9101. 10.1073/pnas.1532872100 PMC166444 12861072

[81] Kuhl PK. Social mechanisms in early language acquisition: Understanding integrated brain systems supporting language In: Decety J, Cacioppo JT, editors. Oxford handbook of social neuroscience. New York: Oxford University Press; 2011 pp. 649–667.

[82] PF3.2: Enrolment in childcare and pre-school. http://www.oecd.org/els/soc/PF3_2_Enrolment_childcare_preschool.pdf [Accessed 19th June 2017].

