# Supplementary material for: Music@Home: A novel instrument to assess the home musical environment in the early years
Source: PLoS One. 2018 Apr 11;13(4):e0193819. doi: 10.1371/journal.pone.0193819 (PMC5894980; doi:10.1371/journal.pone.0193819)
Supplement: S1 Table — (DOCX) [file pone.0193819.s001.docx]

S1 Table. Study1: Music@Home-Preschool: Demographic information for participating parents.

|  | n | % |
| --- | --- | --- |
| **Level of English** |  |  |
| Native | 314 | 90.5% |
| Fluent | 20 | 5.8% |
| Advanced | 13 | 3.7% |
| **Country of Residence** |  |  |
| United Kingdom | 254 | 73.2% |
| United States of America | 25 | 7.2% |
| Australia | 19 | 5.5% |
| Canada | 4 | 1.2% |
| Ireland | 2 | 0.6% |
| New Zealand | 1 | 0.3% |
| Other | 42 | 12.1% |
| **Level of Education** |  |  |
| Did not complete school qualification | 1 | 0.3% |
| First School Qualification (e.g. GCSE/Junior High School) | 11 | 3.2% |
| Second qualification (e.g A levels/ High School) | 32 | 9.2% |
| Undergraduate Degree or professional qualification | 141 | 40.6% |
| Master's degree or above | 162 | 46.7% |
| **Level of Family Income** |  |  |
| £40.000 or lower | 49 | 14.1% |
| £40.000-£60.000 | 80 | 23.1% |
| £60.000-£90.000 | 93 | 26.8% |
| £90.000 or higher | 125 | 36.0% |
