# Supplementary material for: Music@Home: A novel instrument to assess the home musical environment in the early years
Source: PLoS One. 2018 Apr 11;13(4):e0193819. doi: 10.1371/journal.pone.0193819 (PMC5894980; doi:10.1371/journal.pone.0193819)
Supplement: S2 Table — (DOCX) [file pone.0193819.s002.docx]

S2 Table. Study1: Music@Home-Preschool: Demographic information for the respondents’ children.

|  | n | % |
| --- | --- | --- |
| **Gender** |  |  |
| Female | 165 | 47.6% |
| Male | 182 | 52.4% |
| **Language** |  |  |
| English Monolingual | 287 | 82.7% |
| English Bilingual | 27 | 7.8% |
| Monolingual other | 27 | 7.8% |
| Bilingual other | 6 | 1.7% |
| **Number of children in the family** |  |  |
| Only child | 125 | 36.0% |
| 2 children | 169 | 48.7% |
| 3 children | 36 | 10.4% |
| 4 or more children | 17 | 4.9% |
