# Supplementary material for: Music@Home: A novel instrument to assess the home musical environment in the early years
Source: PLoS One. 2018 Apr 11;13(4):e0193819. doi: 10.1371/journal.pone.0193819 (PMC5894980; doi:10.1371/journal.pone.0193819)
Supplement: S3 Table — (DOCX) [file pone.0193819.s003.docx]

S3 Table. Study1: Music@Home-Infant: Demographic information for participating parents.

|  | n | % |
| --- | --- | --- |
| **Level of English** |  |  |
| Native | 255 | 88.9% |
| Fluent | 24 | 8.4% |
| Advanced | 8 | 2.8% |
| **Country of Residence** |  |  |
| United Kingdom | 203 | 70.7% |
| United States of America | 22 | 7.7% |
| Australia | 14 | 4.9% |
| Ireland | 16 | 5.6% |
| Other | 32 | 11.1% |
| **Level of Education** |  |  |
| Did not complete school qualification | 1 | 0.3% |
| First School Qualification (e.g. GCSE/Junior High School) | 6 | 2.1% |
| Second qualification (e.g A levels/ High School) | 30 | 10.5% |
| Undergraduate Degree or professional qualification | 99 | 34.5% |
| Master's degree or above | 151 | 52.6% |
| **Level of Family Income** |  |  |
| £40.000 or lower | 53 | 18.5% |
| £40.000-£60.000 | 73 | 25.4% |
| £60.000-£90.000 | 73 | 25.4% |
| £90.000 or higher | 88 | 30.7% |
|  |  |  |
