# Supplementary material for: Music@Home: A novel instrument to assess the home musical environment in the early years
Source: PLoS One. 2018 Apr 11;13(4):e0193819. doi: 10.1371/journal.pone.0193819 (PMC5894980; doi:10.1371/journal.pone.0193819)
Supplement: S4 Table — (DOCX) [file pone.0193819.s004.docx]

S4 Table. Study1: Music@Home-Infant: Demographic information for the respondents’ children.

|  | n | % |
| --- | --- | --- |
| **Gender** |  |  |
| Female | 155 | 54.0% |
| Male | 132 | 46.0% |
| **Language** |  |  |
| English Monolingual | 224 | 78.0% |
| English Bilingual | 30 | 10.5% |
| Monolingual other | 21 | 7.3% |
| Bilingual other | 10 | 3.5% |
| Trilingual | 2 | 0.7% |
| **Number of children in the family** |  |  |
| Only child | 204 | 71.1% |
| 2 children | 62 | 21.6% |
| 3 children | 11 | 3.8% |
| 4 or more children | 10 | 3.5% |
