# Supplementary material for: Music@Home: A novel instrument to assess the home musical environment in the early years
Source: PLoS One. 2018 Apr 11;13(4):e0193819. doi: 10.1371/journal.pone.0193819 (PMC5894980; doi:10.1371/journal.pone.0193819)
Supplement: S5 Table — (DOCX) [file pone.0193819.s005.docx]

S5 Table. Study1: Structure of factors and item loadings for the Music@Home - Preschool.

| M@H - Preschool Items | M@H-GF | PB | CAE | PIn  MB | BME |
| --- | --- | --- | --- | --- | --- |
|  |  |  |  |  |  |
| 1. I believe that children should learn to play an instrument | .49 | .60 |  |  |  |
| 2. I believe that music is part of a well-rounded education | .44 | .20 |  |  |  |
| 3. My child was deliberately sung to/exposed to music whilst in the womb | .43 | .22 |  |  |  |
| 4. I believe music has an impact on my child's intelligence | .52 | .44 |  |  |  |
| 5. I think musical activities are important for learning to communicate | .51 | .24 |  |  |  |
| 6. My child enjoys making sounds/interacting with musical instruments (including toy ones) | .60 |  | .57 |  |  |
| 7. My child rarely makes music | .58 |  | .45 |  |  |
| 8. My child does not use objects to intentionally produce sounds | .56 |  | .52 |  |  |
| 9. My child enjoys toys with musical features | .46 |  | .37 |  |  |
| 10. I sing in playful contexts to/with my child at least once a day | .57 |  |  | .55 |  |
| 11. I sing to/with my child in many different situations (e.g. during playtime, with friends and family) | .62 |  |  | .48 |  |
| 12. I sing to/with my child several (e.g. 5 - 10) times a day. | .56 |  |  | .70 |  |
| 13. I do not feel comfortable singing to my child in public or when others are around. | .46 |  |  | .23 |  |
| 14. I make music with my child (including toy instruments) almost everyday | .58 |  |  | .49 |  |
| 15. I do not make music with my child (including toy instruments) more than once or twice per week | .59 |  |  | .42 |  |
| 16. My child is exposed to a broad range of musical styles at home (e.g. pop, rap, dance, classical etc) | .53 |  |  |  | .60 |
| 17. I sing all different types of songs to my child (e.g. adult songs, traditional folk songs) | .63 |  |  |  | .53 |
| 18. I would only expose my child to "children's music" | .38 |  |  |  | .43 |
| 19. I sing mostly children's songs or lullabies to or with my child | <.20 |  |  |  | .48 |

Note^1^: M@H-GF = Music@Home-General Factor, PB = Parental Beliefs, CAE = Child’s Active Engagement with Music, PInMB = Parent Initiation of Musical Behaviour, BME = Breadth of Musical Exposure.

Note^2^: Loadings < .20 are not listed.

Note^3:^ Four items with high uniqueness values (>.7) and 27 items with either very low loadings (>.2) or similar loadings on two rather than one factor (loadings between .2 and .4) were removed and the Schmid-Leiman procedure was re-run until 19 items that had adequate loadings on the general and on one of the sub-factors remained.

Note^4^: All items except one had adequate loadings on the general home musical environment factor (loadings >.30) and all items loaded adequately on one of the sub-factors (>.20) and weakly on all the others (<.20). One item that did not adequately load on the general factor was kept because it significantly contributed to one of the sub-factors (item 19, loading = .48) and removing it would weaken this particular sub-factor’s eigenvalue. Please note that the loadings on the sub-factors in this case have lower values than they would have if the general factor had not been accounted for.

Note^4^: Items 12 and 13 were removed after presenting weak loadings on the Parent Initiation of Musical Behaviour factor during the confirmatory factor analysis in Study 1.
