# Supplementary material for: Music@Home: A novel instrument to assess the home musical environment in the early years
Source: PLoS One. 2018 Apr 11;13(4):e0193819. doi: 10.1371/journal.pone.0193819 (PMC5894980; doi:10.1371/journal.pone.0193819)
Supplement: S6 Table — (DOCX) [file pone.0193819.s006.docx]

**S6 Table. Study1: Structure of factors and item loadings for the Music@Home - Infant**

| M@H - Infant Items | M@H-GF | PB | ER | CAE | PInS | PInMM |
| --- | --- | --- | --- | --- | --- | --- |
|  |  |  |  |  |  |  |
| 1. I believe that children should learn to play an instrument | .46 | .50 |  |  |  |  |
| 2. I believe that music is part of a well rounded education | .48 | .60 |  |  |  |  |
| 3. My child was deliberately sung to/exposed to music whilst in the womb | .44 | .49 |  |  |  |  |
| 4. I believe music has an impact on my child's intelligence | .46 | .46 |  |  |  |  |
| 5. I sing to soothe my child | .44 |  | .37 |  | *.21* |  |
| 6. I find music does not influence my child's mood or emotional state | .44 |  | .42 |  |  |  |
| 7. I find my child is not soothed by music or singing | .41 |  | .76 |  |  |  |
| 8. My child displays no physical signs of engagement when there is recorded music on (e.g. bouncing or tapping) | .37 |  | *.22* | .61 |  |  |
| 9. I encourage my child to move along to music | .43 |  |  | .46 |  |  |
| 10. I have noticed my child moving in time with the beat of the music | .25 |  |  | .74 |  |  |
| 11. My child does not dance/move to music on the stereo or television | .32 |  |  | .87 |  |  |
| 12. Music does not evoke a physical response from my child | .45 |  |  | .72 |  |  |
| 13. My child rarely makes music | .47 |  |  | .48 |  | *.29* |
| 14. 1 sing in playful contexts to/with my child at least once a day | .64 |  |  |  | .47 |  |
| 15. I sing to/with my child several (e.g. 5 - 10) times a day | .66 |  |  |  | .50 |  |
| 16. I teach my child new songs | .60 |  |  |  | .37 |  |
| 17. I do not usually choose to play games that involve singing/music with my child | .58 |  |  |  | .29 |  |
| 18. I sing to/with my child in many different situations (e.g. during playtime, with friends and family) | .67 |  |  |  | .41 |  |
| 19. During our daily routine, I do not spend much time singing about what we are doing | .56 |  |  |  | .43 |  |
| 20. Our daily routines often involve music (e.g. during tooth brushing, bath time) | .58 |  |  |  | .21 |  |
| 21. Making music with my child (including toy instruments) is a regular part of playtime at home | .63 |  |  |  |  | .54 |
| 22. I make music with my child (including toy instruments) almost everyday | .67 |  |  |  |  | .55 |
| 23. I do not make music with my child (including toy instruments) more than once or twice per week | .67 |  |  |  |  | .45 |

Note^1^: M@H-GF = Music@Home-General Factor, PB = Parental Beliefs, ER = Emotion Regulation CAE = Child’s Active Engagement with Music, PInS = Parent Initiation of Singing, PInMM = Parent Initiation of Music Making.

Note^2^: Loadings < .20 are not listed.

Note^3^: Twenty out of 23 items had high loadings on the general home musical environment factor (>.40). The 3 items with weaker loadings on the general factor had very high loadings on one of the sub-factors (>.60). All items loaded adequately on one of the sub-factors (>.20). Three items that were loading on two sub-factors rather than one were kept because [a] their loading on one factor was much higher than the other [b] they were conceptually relevant to the factors they loaded more highly on, and [c] removing them would weaken the respective factors’ eigenvalues.

Note^4^: Items 17 and 20 were removed after presenting weak loadings on the Parent Initiation of Singing factor during the confirmatory factor analysis in Study 1.
