# Supplementary material for: Music@Home: A novel instrument to assess the home musical environment in the early years
Source: PLoS One. 2018 Apr 11;13(4):e0193819. doi: 10.1371/journal.pone.0193819 (PMC5894980; doi:10.1371/journal.pone.0193819)
Supplement: S7 Table — (DOCX) [file pone.0193819.s007.docx]

S7 Table. Study 2: Music@Home-Preschool: Demographic information for participating parents.

|  | n | % |
| --- | --- | --- |
| **Level of English** |  |  |
| Native | 183 | 85.9% |
| Fluent | 15 | 7.0% |
| Advanced | 15 | 7.0% |
| **Country of Residence** |  |  |
| United Kingdom | 163 | 76.5% |
| United States of America | 21 | 9.9.% |
| Australia | 6 | 2.8% |
| Canada | 1 | 0.5% |
| Ireland | 2 | 0.9% |
| New Zealand | 1 | 0.5% |
| Other | 19 | 8.9% |
| **Level of Education** |  |  |
| Did not complete school qualification | - | 0% |
| First School Qualification (e.g. GCSE/Junior High School) | 4 | 1.9% |
| Second qualification (e.g A levels/ High School) | 8 | 3.8% |
| Undergraduate Degree or professional qualification | 65 | 30.5% |
| Master's degree or above | 136 | 63.8% |
| **SES (NS-SEC)** |  |  |
| Managerial and professional occupations | 191 | 89.7% |
| Intermediate occupations | 7 | 3.3% |
| Small employers and own account workers | 10 | 4.7% |
| Lower supervisory and technical occupations | 2 | 0.9% |
| Semi-routine and routine occupations | 3 | 1.4% |
