# Supplementary material for: Music@Home: A novel instrument to assess the home musical environment in the early years
Source: PLoS One. 2018 Apr 11;13(4):e0193819. doi: 10.1371/journal.pone.0193819 (PMC5894980; doi:10.1371/journal.pone.0193819)
Supplement: S8 Table — (DOCX) [file pone.0193819.s008.docx]

S8 Table. Study 2: Music@Home-Preschool: Demographic information for the respondents’ children.

|  | n | % |
| --- | --- | --- |
| **Gender** |  |  |
| Female | 84 | 39.4% |
| Male | 129 | 60.6% |
| **Language** |  |  |
| English Monolingual | 186 | 87.3% |
| English Bilingual | 14 | 6.6% |
| Monolingual other | 11 | 5.2% |
| Bilingual other | 2 | 0.9% |
| **Number of children in the family** |  |  |
| Only child | 99 | 46.5% |
| 2 children | 90 | 42.3% |
| 3 children | 16 | 7.5% |
| 4 or more children | 8 | 3.8% |
