# Supplementary material for: Music@Home: A novel instrument to assess the home musical environment in the early years
Source: PLoS One. 2018 Apr 11;13(4):e0193819. doi: 10.1371/journal.pone.0193819 (PMC5894980; doi:10.1371/journal.pone.0193819)
Supplement: S9 Table — (DOCX) [file pone.0193819.s009.docx]

S9 Table. Study 2: Demographic information for Music@Home-Infant participating parents.

|  | n | % |
| --- | --- | --- |
| **Level of English** |  |  |
| Native | 187 | 87.8% |
| Fluent | 15 | 7.0% |
| Advanced | 11 | 5.2% |
| **Country of Residence** |  |  |
| United Kingdom | 183 | 85.9% |
| United States of America | 9 | 4.2% |
| Canada | 2 | 0.9% |
| Australia | 3 | 1.4% |
| Ireland | 1 | 0.5% |
| Other | 15 | 7.0% |
| **Level of Education** |  |  |
| First School Qualification (e.g. GCSE/Junior High School) | 10 | 4.7% |
| Second qualification (e.g A levels/ High School) | 7 | 3.3% |
| Undergraduate Degree or professional qualification | 85 | 39.9% |
| Master's degree or above | 109 | 51.2% |
| Missing | 2 | 0.9% |
| **SES (NS-SEC)** |  |  |
| Managerial and professional occupations | 185 | 86.9% |
| Intermediate occupations | 8 | 3.8% |
| Small employers and own account workers | 10 | 4.7% |
| Lower supervisory and technical occupations | 4 | 1.9% |
| Semi-routine and routine occupations | 5 | 2.3% |
| Missing | 1 | 0.5% |
