# Supplementary material for: Music@Home: A novel instrument to assess the home musical environment in the early years
Source: PLoS One. 2018 Apr 11;13(4):e0193819. doi: 10.1371/journal.pone.0193819 (PMC5894980; doi:10.1371/journal.pone.0193819)
Supplement: S10 Table — (DOCX) [file pone.0193819.s010.docx]

S10 Table. Study 2: Music@Home-Infant: Demographic information for the respondents’ children.

|  | n | % |
| --- | --- | --- |
| **Gender** |  |  |
| Female | 108 | 50.7% |
| Male | 105 | 49.3% |
| **Language** |  |  |
| English Monolingual | 176 | 82.6% |
| English Bilingual | 16 | 7.5% |
| Monolingual other | 17 | 8.0% |
| Bilingual other | 4 | 1.9% |
| **Number of children in the family** |  |  |
| Only child | 130 | 61.0% |
| 2 children | 62 | 29.1% |
| 3 children | 17 | 8.0% |
| 4 or more children | 4 | 1.9% |
