# Supplementary material for: Music@Home: A novel instrument to assess the home musical environment in the early years
Source: PLoS One. 2018 Apr 11;13(4):e0193819. doi: 10.1371/journal.pone.0193819 (PMC5894980; doi:10.1371/journal.pone.0193819)
Supplement: S1 Fig — (PDF) [file pone.0193819.s011.pdf]

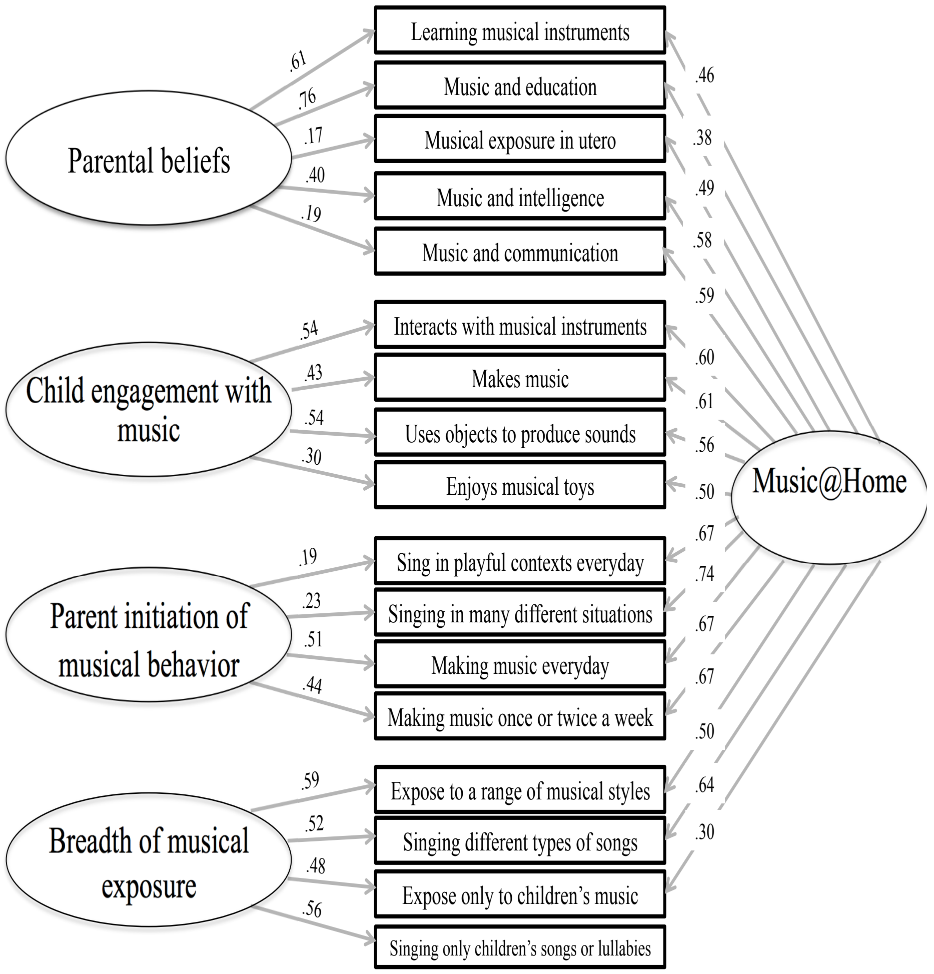

S1 Fig. Study 1: Factor structure of the Music@Home-Preschool as formalized by confirmatory factor analysis.
